# Serum Asprosin Concentrations and Severity in Acute Pancreatitis: A Prospective Case–Control Study with Serial Measurements

**DOI:** 10.3390/diagnostics16213700

**Published:** 2026-11-09

**Authors:** Mehmet Fatih Uzanulu, Musa Ak, Levent Bolat, Onur Selçuk Yiğit, Cihat Uçar, Hacı Mehmet Çitil, Handan Kanat Seyhan

**Affiliations:** 1 Department of Internal Medicine, Malatya Turgut Ozal University Training and Research Hospital, 44430 Malatya, Türkiye; mehmet4436@gmail.com (H.M.Ç.); handankanat@hotmail.com (H.K.S.); 2 Division of Hematology, Department of Internal Medicine, Istanbul Faculty of Medicine, Istanbul University, 34093 Istanbul, Türkiye; musa.ak@istanbul.edu.tr; 3 Department of General Surgery, Osmaniye Training and Research Hospital, 80010 Osmaniye, Türkiye; levent_268@hotmail.com; 4 Department of Internal Medicine, Ordu State Hospital, 52200 Ordu, Türkiye; onursyigit@gmail.com; 5 Department of Physiology, Faculty of Medicine, Malatya Turgut Ozal University, 44210 Malatya, Türkiye; cihat.ucar@ozal.edu.tr

**Keywords:** acute pancreatitis, asprosin, adipokine, biomarker, disease severity, revised Atlanta classification

## Abstract

**Background/Objectives:** Asprosin is a fasting-induced glucogenic adipokine with pro-inflammatory effects in experimental models, but its behaviour in acute pancreatitis (AP) is unknown. In this exploratory study, we examined whether circulating asprosin differs between patients with AP and healthy controls, how it changes during hospitalisation and whether it is associated with disease severity. **Methods:** In this prospective case–control study, 82 patients with AP (of 183 screened, after exclusion of diabetes, hypertriglyceridaemia and morbid obesity) and 82 sex-matched healthy controls were enrolled. Serum asprosin was measured by ELISA at admission, 72 h and 120 h in patients and once in fasting controls. Severity was defined by the revised Atlanta classification. Because patients were older and heavier than controls, the group difference was adjusted for age, body mass index and sex and examined in post hoc matched subsamples. **Results:** Admission asprosin was 4.52 (2.94–5.89) ng/mL in patients and 3.55 (2.24–4.92) ng/mL in controls (*p* = 0.071); the ratio of geometric means adjusted for age, body mass index and sex was 1.17 (95% CI 0.85–1.61). Asprosin was higher in patients in post hoc age- and sex-matched pairs (median difference 1.91 ng/mL, 95% CI 0.24–3.78) but not in body mass index- and sex-matched pairs (0.96 ng/mL, −0.34 to 2.72). Asprosin changed little over five days and was not associated with moderately severe or severe AP (AUC 0.57, upper 95% confidence limits ≤0.71); only seven patients had severe AP. **Conclusions:** In this exploratory study, serum asprosin was modestly and non-significantly higher in patients with AP than in controls, and no statistically significant association with severity was detected. Residual confounding by age and body mass index, the exclusion of patients with diabetes or hypertriglyceridaemia (≥200 mg/dL) and the absence of in-house assay validation preclude firm conclusions. These data provide effect estimates for adequately powered, matched studies of asprosin in AP.

## 1. Introduction

Acute pancreatitis (AP) is a common inflammatory disorder of the pancreas. Hospital admissions for AP have risen markedly [1], and overall mortality is now approximately 2% [2]; however, about 20% of patients develop moderately severe or severe disease, in whom mortality is considerably higher [2,3].

Early prediction of severity therefore has direct clinical value. Numerous clinical, radiological and laboratory predictors have been developed [2,4]; some, such as the Ranson score and C-reactive protein (CRP), require 48–72 h [5,6], whereas indices derived from the complete blood count, such as the neutrophil-to-lymphocyte ratio (NLR), are immediately available [7,8]. The reference standard against which such markers are evaluated is of critical importance.

Asprosin, a 140-amino-acid C-terminal cleavage product of profibrillin, was identified in 2016 as a fasting-induced adipokine that increases hepatic glucose release [9] and acts centrally as an orexigenic hormone [10]. Circulating concentrations are elevated in insulin resistance and type 2 diabetes mellitus [11,12], as reviewed elsewhere [13,14].

Asprosin also has pro-inflammatory actions. Through Toll-like receptor 4, it induces inflammatory signalling in pancreatic β-cells [15] and cytokine release from THP-1 macrophages [16], it increases cytokine production in adipocyte–macrophage co-cultures [17], and it activates the IKKβ–NF-κBp65 axis in endothelium [18] and the NLRP3 inflammasome in models of hypertension [19]. Clinical observations are discordant: serum asprosin was elevated in a small series of post-ERCP pancreatitis [20], lower during attacks of familial Mediterranean fever [21] and higher in sepsis [22]. No study has examined asprosin with serial sampling in community-acquired AP.

We therefore conducted an exploratory prospective case–control study to determine whether circulating asprosin differs between patients with AP and healthy controls and how it changes over the first five days of hospitalisation. Secondary, hypothesis-generating aims were to compare aetiologies and to examine whether asprosin is associated with moderately severe or severe disease as defined by the revised Atlanta classification.

## 2. Materials and Methods

### 2.1. Study Design and Setting

This prospective case–control study was conducted in the Department of Internal Medicine of Malatya Turgut Ozal University Training and Research Hospital between 5 December 2025 and 1 August 2026, with the approval of the institutional ethics committee (2025/439) and written informed consent from all participants. It is reported according to the STROBE statement and, for the diagnostic accuracy component, the STARD statement [23,24].

### 2.2. Participants

**Figure 1:**
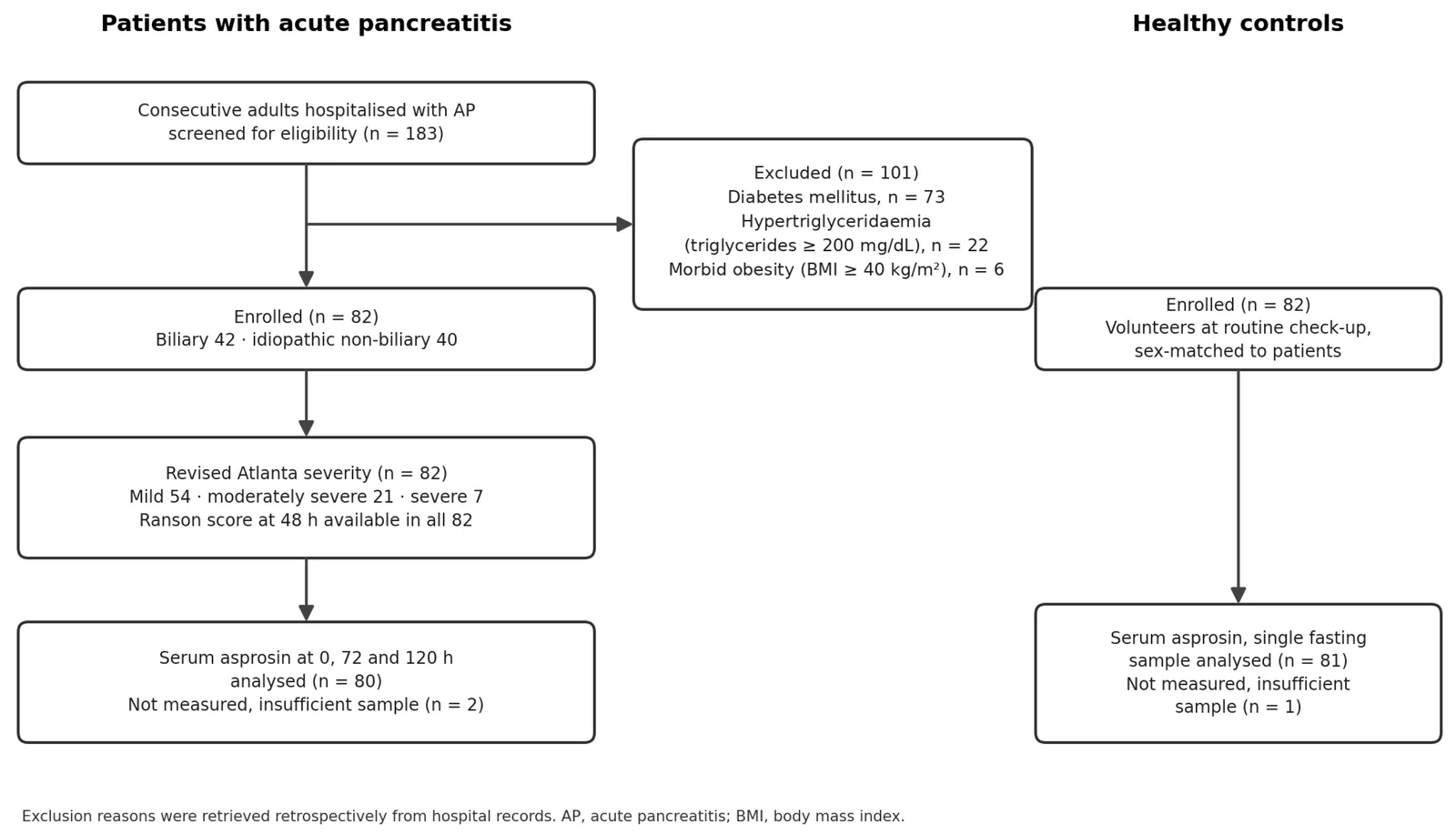
Study flow diagram of patients with acute pancreatitis and healthy controls.

Consecutive adults (≥18 years) hospitalised with AP, diagnosed by at least two revised Atlanta criteria [3], were screened. AP was classified as biliary when gallstones or sludge were demonstrated on ultrasonography or computed tomography; non-biliary cases without alcohol use or another identified cause were classified as idiopathic. Because circulating asprosin is elevated in insulin resistance, diabetes, dyslipidaemia and obesity [11,12,13,14], patients with diabetes mellitus, prediabetes (glycated haemoglobin [HbA1c] 5.7–6.4%), hypertriglyceridaemia (triglycerides ≥ 200 mg/dL, regardless of pancreatitis aetiology) or morbid obesity (body mass index ≥ 40 kg/m^2^) were excluded; malignancy, chronic pancreatitis, infection at another site, chronic inflammatory or autoimmune disease and pregnancy were further exclusion criteria. Individual reasons for exclusion were not recorded prospectively; they were retrieved retrospectively from the hospital records during revision of this manuscript and are reported in Section 3.1 and Figure 1.

Controls were volunteers attending the routine check-up clinic of the same hospital, with no diagnosis of pancreatitis, diabetes mellitus, hyperlipidaemia, inflammatory disease, atherosclerotic heart disease or insulin resistance; HbA1c was not measured in controls. Controls were matched to patients for sex only. Matching for age and body mass index at the design stage was not feasible: most patients were elderly, and healthy volunteers over 60 years of age without diabetes, dyslipidaemia or atherosclerotic disease were rare in the check-up population from which controls were recruited: in the nationwide TURDEP-II survey, the prevalence of diabetes (16.5%) and prediabetes (30.8%) in Turkish adults rises steeply with age [25]. Such matching would therefore have excluded most older patients or greatly reduced the number of controls. We therefore accepted sex matching and addressed age and body mass index by statistical adjustment and post hoc matching (Section 2.6). This is a limitation of the design, and residual confounding cannot be excluded. Because controls were selected on recorded diagnoses rather than laboratory criteria, a sensitivity analysis excluded the 25 controls with fasting glucose ≥ 100 mg/dL or triglycerides ≥ 200 mg/dL.

Of the 183 patients screened, 82 met the eligibility criteria and were included together with 82 controls (Figure 1). Asprosin could not be measured in 2 patients and 1 control because of insufficient sample volume; asprosin analyses therefore include 80 patients and 81 controls.

### 2.3. Clinical and Laboratory Data

Demographic and anthropometric data, smoking, alcohol use, time to oral refeeding and length of stay were recorded prospectively. Laboratory tests included complete blood count, amylase, lipase, CRP, erythrocyte sedimentation rate (ESR), albumin, total protein, lactate dehydrogenase, liver enzymes, creatinine, urea, glucose, insulin (for the homeostasis model assessment of insulin resistance, HOMA-IR), albumin-corrected calcium and lipids. The NLR and CRP-to-albumin ratio were calculated, and CRP and the ESR were repeated at 72 h and 120 h.

### 2.4. Blood Sampling and Measurement of Asprosin

In accordance with the routine practice of our department, all patients were kept nil by mouth with intravenous fluid hydration for at least 48 h, and oral refeeding was initiated after 48 h or 72 h, guided by clinical improvement and resolution of abdominal pain. This practice was not a requirement of the study, which did not influence clinical management. Time from symptom onset to admission and the duration of pre-admission fasting were not recorded; the admission sample (0 h) therefore reflected the presenting state before in-hospital fasting and hydration. The 72 h and 120 h samples were obtained after oral refeeding in most or all patients. Control subjects were sampled once after an overnight fast of at least 12 h.

Venous blood samples were obtained at admission (0 h), 72 h and 120 h in patients, collected into plain tubes without anticoagulant, allowed to clot at room temperature and centrifuged at 1500× *g* for 15 min at +4 °C. The separated serum was immediately aliquoted and stored at −80 °C. Samples underwent a single freeze–thaw cycle immediately before the assay, and none showed visible haemolysis or lipaemia.

Serum asprosin concentrations were quantified using a commercial sandwich enzyme-linked immunosorbent assay (ELISA) kit (Cat. No. E4095Hu, Lot No. 202606013, Bioassay Technology Laboratory, Shanghai, China) following the manufacturer’s protocol; reagents and pre-coated plates from this single production lot were used for all runs. The manufacturer-reported nominal assay range was 0.5–100 ng/mL, with an analytical sensitivity of 0.23 ng/mL. The eight-point standard curve calibrators were assayed in duplicate on every plate (R^2^ > 0.99 on all plates; coefficient of variation [CV] of duplicate calibrators < 5%). Study samples were analysed in singlicate because of limited serum volume. To avoid inter-assay variation in longitudinal comparisons, all serial samples from a given patient (0, 72 and 120 h) were assayed on the same microplate, and patient and control samples were randomly interspersed across plates. Measured concentrations ranged from 0.38 to 65.0 ng/mL; none of the 321 measurements exceeded the upper limit of the nominal range, and 1 (a control sample, 0.38 ng/mL) was below its lower limit but above the analytical sensitivity. The manufacturer-reported intra-assay and inter-assay CVs were <10% and <12%, respectively, and the internal kit quality controls run on each plate fell within acceptable limits. No in-house validation of the assay (duplicate precision of study samples, dilution linearity or spike recovery) was performed. Laboratory personnel performing the assays were blinded to group allocation, clinical severity and patient outcomes.

### 2.5. Definitions of Severity and Outcome Measures

The primary outcome was the difference in admission (0 h) asprosin between patients and controls. Pre-specified secondary outcomes were the change in asprosin over time and its association with moderately severe or severe disease by the revised Atlanta classification. All other analyses (aetiological comparisons, the severe category alone, correlations and the adjusted and matched comparisons introduced to address confounding, together with Ranson-based sensitivity analyses) were exploratory or post hoc; Table 1 separates pre-specified from post hoc analyses. Because the time at which organ failure and complications developed was not recorded, asprosin at 72 h and 120 h was treated as a concurrent correlate of severity and not as an early predictor.

Severity was classified by the revised Atlanta classification [3], with organ failure assessed by the modified Marshall score [3,26]: mild (no organ failure or complications), moderately severe (transient organ failure ≤ 48 h and/or local or systemic complications) and severe (persistent organ failure > 48 h). Because only seven patients had severe AP, analyses of that category are descriptive.

A Ranson score ≥ 3 at 48 h was used as a secondary criterion in a post hoc sensitivity analysis, and agreement with the revised Atlanta classification was assessed with the Cohen kappa coefficient.

### 2.6. Statistical Analysis

Analyses were performed in Python 3.11 (SciPy 1.17, scikit-learn 1.8 and statsmodels 0.15). Because asprosin and most variables were non-normally distributed (Shapiro–Wilk *p* < 0.001), non-parametric methods were used, and data are presented as the median (interquartile range).

Two groups were compared with the Mann–Whitney U test (effect size: rank-biserial r with bootstrap 95% confidence interval [CI]) and three groups with the Kruskal–Wallis test followed by Bonferroni-corrected Dunn tests; trend across Atlanta categories was tested with the Jonckheere–Terpstra test and categorical variables with the chi-square test. Serial data were analysed with the Friedman test (Kendall W), Bonferroni-corrected Wilcoxon tests and a linear mixed-effects model with a random patient intercept on rank-transformed values. The 84 Spearman correlations of asprosin were adjusted with the Benjamini–Hochberg procedure; partial correlations of asprosin with length of stay were a separate, unadjusted exploratory analysis.

Because the patients were older and had a higher body mass index than the controls, the group difference was re-examined in three ways. First, log-transformed asprosin was regressed on group, age, body mass index and sex with heteroscedasticity-robust standard errors, and the result is expressed as the ratio of geometric means (patients to controls) with 95% CI; the unadjusted Hodges–Lehmann median difference is also reported. Second, as sensitivity analyses, rank-based regression, logarithmic transformation, trimming of extreme values and exclusion of metabolically ineligible controls were applied (Supplementary Table S1). Third, in post hoc analyses, patients and controls were matched 1:1 without replacement by optimal matching on sex and age (±3 years), on sex and body mass index (±1 kg/m^2^) or on all three, and pairs were compared with the Wilcoxon signed-rank test and the Hodges–Lehmann median paired difference. Equivalence was tested post hoc with two one-sided tests (TOST) on log-transformed values with a margin of ±0.5 standardised units, a medium effect, because no minimal clinically important difference exists for asprosin.

Discrimination was assessed by receiver operating characteristic (ROC) analysis with bootstrap 95% CI for the area under the curve (AUC); paired AUCs (the ESR vs. CRP) were compared with the DeLong test. With 28 events, multivariable modelling was not performed. Apart from asprosin, there were no missing data, and analyses were complete-case. A two-sided *p* < 0.05 was considered significant.

## 3. Results

### 3.1. Participant Characteristics and Disease Severity

Of the 183 patients screened, 101 (55%) were excluded: 73 because of diabetes mellitus, 22 because of hypertriglyceridaemia (triglycerides ≥ 200 mg/dL) and 6 because of morbid obesity; no patient was excluded for the remaining criteria (Figure 1). The cohort therefore represents AP in patients without diabetes, hypertriglyceridaemia (≥200 mg/dL) or morbid obesity. All 40 non-biliary cases were idiopathic, and no patient had alcohol-related AP. Oral refeeding was started after 48 h in 57 patients (69.5%) and after 72 h in 25 (30.5%), with a similar distribution in biliary and non-biliary AP (29 of 42 vs. 28 of 40).

The sex distribution was balanced (45.1% vs. 42.7% men; *p* = 0.875), but patients were older than controls (median 62 [44–76] vs. 42 [32–52] years; *p* < 0.001) and had a higher body mass index (27.27 vs. 24.67 kg/m^2^; *p* < 0.001) (Table 2). As expected in acute illness, HOMA-IR (2.74 [2.31–3.42] vs. 1.93 [1.69–2.26]) and glucose (94 vs. 90 mg/dL) were higher and triglycerides lower (89 vs. 130 mg/dL) in patients (all *p* < 0.001).

Fifty-four patients (65.9%) had mild, twenty-one (25.6%) moderately severe and seven (8.5%) severe AP; all severe cases had persistent respiratory failure. Local complications occurred in twenty patients (24.4%) and systemic complications in five (6.1%); two patients required intensive care, and none died. Severity did not differ by aetiology (*p* = 0.742).

### 3.2. Serum Asprosin in Patients and Controls

Twenty-two of the twenty-five laboratory parameters differed between groups (Table 2), with complete separation for amylase and lipase (r = 1.00) and large effect sizes for albumin (r = −0.77), the CRP-to-albumin ratio (0.74), lactate dehydrogenase (0.71), CRP (0.70) and the NLR (0.69).

**Figure 2:**
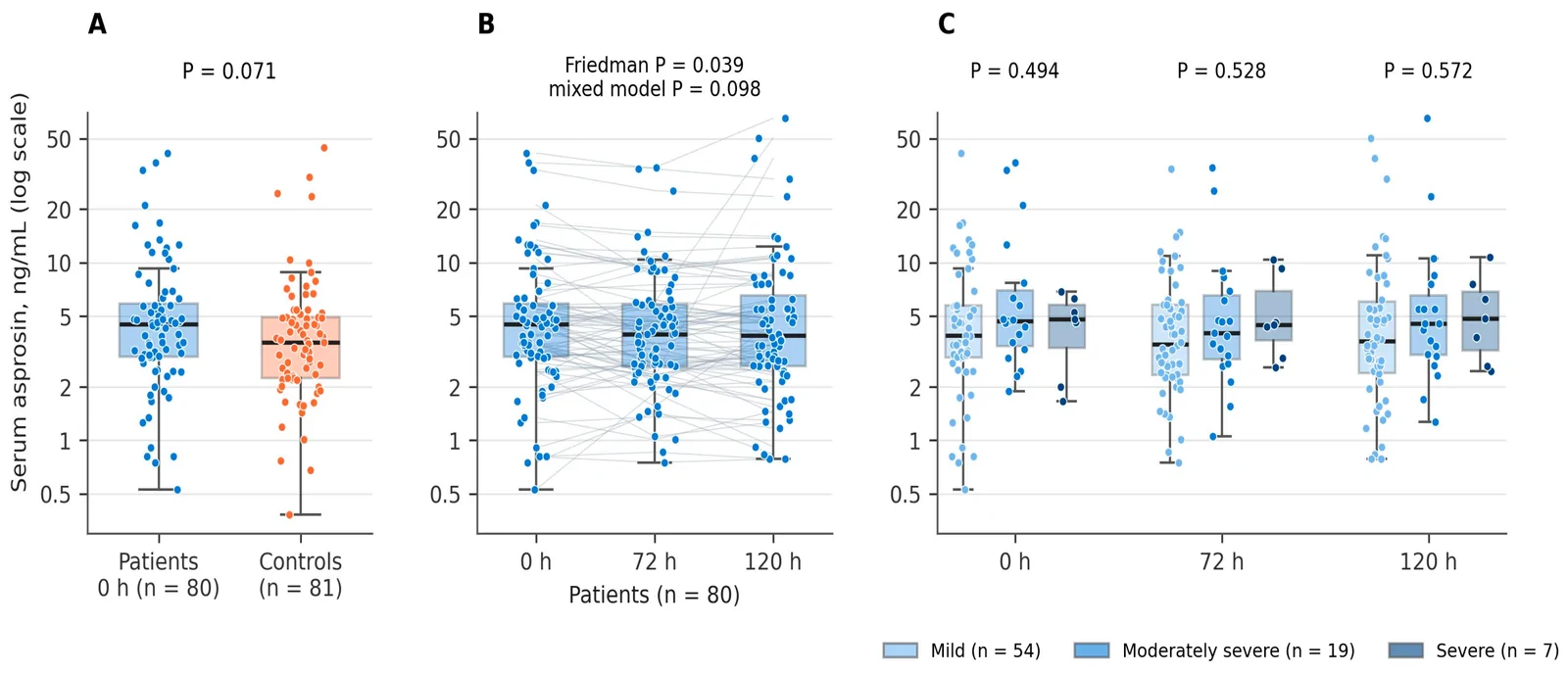
Serum asprosin concentrations by study group, sampling time and acute pancreatitis severity. (**A**) Admission values in patients with acute pancreatitis and healthy controls. (**B**) Serial values at admission, 72 h and 120 h in patients. (**C**) Values at each sampling time according to the revised Atlanta severity categories.

Admission asprosin was 4.52 (2.94–5.89) ng/mL in patients (*n* = 80) and 3.55 (2.24–4.92) ng/mL in controls (*n* = 81) (*p* = 0.071; r = 0.16, 95% CI −0.01 to 0.34; Figure 2A). The Hodges–Lehmann median difference was 0.70 ng/mL (95% CI −0.06 to 1.42) and the unadjusted ratio of geometric means 1.21 (0.95–1.56). Equivalence within ±0.5 standardised units was not demonstrated either (standardised difference 0.24, 90% CI −0.02 to 0.50; TOST *p* = 0.051). Apart from asprosin, only albumin-corrected calcium (*p* = 0.296) and LDL cholesterol (*p* = 0.053) did not differ between groups.

After adjustment for age, body mass index and sex, the ratio of geometric means was 1.17 (95% CI 0.85–1.61; *p* = 0.324) and, after exclusion of metabolically ineligible controls, 1.23 (0.85–1.77); rank-based regression, logarithmic transformation and trimming gave *p* values of 0.062–0.128 (Supplementary Tables S1 and S11). Age was not associated with asprosin in either group (group × age interaction *p* = 0.162). In the post hoc matched subsamples, asprosin was higher in patients than in controls when matched for age and sex (46 pairs; median paired difference 1.91 ng/mL, 95% CI 0.24 to 3.78; *p* = 0.022) and when matched for age, body mass index and sex (23 pairs; 2.27 ng/mL, 0.05 to 5.21; *p* = 0.042) but not when matched for body mass index and sex (45 pairs; 0.96 ng/mL, −0.34 to 2.72; *p* = 0.150). The three estimates were in the same direction with overlapping confidence intervals. The matched subsamples retained only 29–58% of patients, mainly the younger and leaner ones (age matching excluded 34 patients with a median age of 77 years), and these analyses were not pre-specified or corrected for multiplicity.

### 3.3. Serial Changes During Hospitalisation

Asprosin changed little during hospitalisation (*n* = 80; 4.52 → 3.95 → 3.88 ng/mL; Friedman *p* = 0.039; Kendall W = 0.041; Figure 2B). Only the 0–72 h comparison was significant after correction (*p* = 0.018), and in the mixed model accounting for within-patient correlation, the overall effect of time was not significant (likelihood-ratio *p* = 0.098; Supplementary Table S10). The change did not differ by time of refeeding (*p* = 0.883; time × refeeding interaction *p* = 0.617).

By comparison, CRP and the ESR peaked at 72 h and declined by 120 h (CRP 8.75 → 65.30 → 38.65 mg/L; ESR 19 → 31 → 22 mm/h; both *p* < 0.001). The ESR discriminated patients from controls as well as CRP did at all time points (AUC 0.947 vs. 0.950 at 72 h; DeLong *p* = 0.906) but did not differ across Atlanta categories (*p* = 0.587–0.960); the two markers were only moderately correlated (ρ = 0.20–0.41).

### 3.4. Biliary and Non-Biliary Aetiology

Cholestatic enzymes were higher in biliary than in non-biliary AP (corrected *p* ≤ 0.012), supporting the aetiological classification (Supplementary Table S2). After correction, asprosin did not differ from controls in either subgroup (biliary corrected *p* = 1.000; non-biliary 0.102–0.136; Kruskal–Wallis *p* = 0.078–0.128); nominal differences in the non-biliary subgroup (unadjusted *p* = 0.031–0.044) disappeared after adjustment for its older age and higher body mass index (Supplementary Table S3).

### 3.5. Disease Severity and Diagnostic Performance

Moderately severe or severe disease occurred in 28 patients (34.1%), of whom only seven had severe AP; the severity analyses are therefore exploratory. Asprosin did not differ across Atlanta categories (*p* = 0.494–0.572; trend *p* = 0.272–0.312; Table 3A, Figure 2C) or between moderately severe or severe and mild disease (*p* = 0.276–0.299; Supplementary Table S4); only age increased across categories (*p* = 0.008).

Age discriminated best (AUC 0.653; 95% CI 0.525–0.771), followed by CRP at 120 h (0.640) and the Ranson score (0.638) (Table 3B, Supplementary Figure S1). Asprosin AUCs were 0.576, 0.573 and 0.572 at 0, 72 and 120 h, with all 95% CI including 0.50 (*p* = 0.276–0.299); the NLR was also non-discriminative (AUC 0.560).

In the post hoc sensitivity analysis using a Ranson score ≥ 3 at 48 h (23 patients, 28.0%), agreement with the Atlanta classification was only fair (kappa = 0.235, 95% CI −0.001 to 0.471; Supplementary Table S5). Asprosin did not differ between Ranson strata (*p* = 0.284–0.738), whereas the NLR did (*p* < 0.001; Supplementary Table S6).

### 3.6. Correlation Analyses

None of the 84 Spearman correlations of asprosin survived Benjamini–Hochberg adjustment (all q ≥ 0.16; Supplementary Tables S7–S9). Nominally, asprosin correlated inversely with glucose (ρ = −0.231 to −0.295) and with length of stay (ρ = −0.238 to −0.273; partial ρ adjusted for age, Atlanta category and time to refeeding −0.244 to −0.315) at all three time points.

## 4. Discussion

In this exploratory prospective study with serial sampling, serum asprosin was modestly higher in patients with AP than in healthy controls, but the difference was not statistically significant in the primary analysis or after adjustment for age, body mass index and sex and reached significance only in post hoc matched subsamples. Asprosin changed little over five days, did not differ by aetiology and showed no statistically significant association with severity by the revised Atlanta classification. Because of the design limitations discussed below, these findings neither establish nor exclude a role of asprosin in AP. They indicate that any systemic response of circulating asprosin to acute pancreatic inflammation is at most modest, and they provide effect estimates for planning adequately powered studies.

The patient–control comparison was pathophysiological rather than diagnostic because AP is readily diagnosed from symptoms, enzymes and imaging; its purpose was to test whether circulating asprosin responds to pancreatic inflammation, as its experimental pro-inflammatory actions [15,16,17,18,19] might predict. The clinically relevant question, early prediction of severity, could be addressed only by the admission measurement; the later measurements describe concurrent associations.

### 4.1. Confounding, Selection and Analytical Considerations

The principal limitation is the case–control design. Controls were matched for sex only and were about 20 years younger and 2.6 kg/m^2^ leaner than patients because healthy volunteers over 60 years of age without diabetes, dyslipidaemia or atherosclerotic disease were rare in the check-up population from which controls were recruited. Age was not associated with asprosin in either group and body mass index only weakly, but statistical adjustment and post hoc matching cannot fully correct imbalances of this size, and residual confounding cannot be excluded. This applies to the negative primary result as much as to the nominally positive matched analyses, which retained mainly the younger and leaner patients. Second, 101 out of 183 screened patients (55%) were excluded, mainly because of diabetes (*n* = 73) or hypertriglyceridaemia (*n* = 22). These exclusions were necessary because both conditions raise circulating asprosin [11,12] and would have confounded the comparison, but they mean that the cohort represents AP without diabetes, hypertriglyceridaemia (≥200 mg/dL) or morbid obesity, and the findings should not be generalised to the full spectrum of AP.

Third, twenty-two of the twenty-five laboratory parameters measured in the same samples separated the groups, which supports the integrity of sample collection, processing and storage but does not validate the asprosin assay itself. Study samples were measured in singlicate with a commercial ELISA without in-house validation of precision, dilution linearity or recovery, and reported asprosin concentrations differ by orders of magnitude between published studies (Section 4.3). An analytical contribution to our findings therefore cannot be excluded, and the results should not be taken as establishing the absence of an association.

### 4.2. Choice of Reference Standard

Agreement between a Ranson score ≥ 3 and the revised Atlanta classification was only fair (kappa = 0.235). The Ranson score predates the revised classification, relies on laboratory variables and is no longer considered adequate on its own [3,4].

In this cohort, the NLR was associated with the Ranson criterion (*p* < 0.001) but not with Atlanta severity (AUC 0.560), possibly because both the Ranson score and the NLR incorporate leucocyte counts. Given the fair agreement and modest numbers, this observation should not be generalised beyond our data. For asprosin, the result was negative under both criteria.

### 4.3. Asprosin in Acute Pancreatitis and Other Inflammatory States

Our findings differ from the only previous pancreatitis report, in which asprosin was higher in 25 patients with post-ERCP pancreatitis than in 21 controls [20]. Post-ERCP pancreatitis is an iatrogenic, temporally defined injury in patients fasted in a standardised manner, and that study was small; its inverse correlations of asprosin with leucocyte and lymphocyte counts are, moreover, compatible with our failure to find a positive association with inflammatory activity.

Visceral adipose tissue is thought to amplify the systemic response in AP, which provides a rationale for studying adipokines [27,28]. The evidence has, however, been largely negative: in a 2024 meta-analysis of adipokines, only resistin was higher in severe AP (standardised mean difference 0.78), whereas leptin, adiponectin and visfatin were not [27], and in a prospective cohort, only resistin and interleukin-6 predicted severe disease [28]. Resistin is secreted by macrophages and behaves as part of the acute-phase response, whereas asprosin is a nutritional signal regulated by fasting [9,10].

Asprosin has been reported to fall during attacks of familial Mediterranean fever [21] and has been examined in sepsis [22], whereas it is elevated in chronic conditions such as dilated cardiomyopathy [29], coronary artery disease (reported in μg/mL, compared with ng/mL in our study) [30] and metabolic syndrome [31]. One possible pattern, based on few heterogeneous studies using different assays, is that asprosin rises in chronic, metabolically driven low-grade inflammation but not in acute systemic inflammation.

The small decline between 0 and 72 h coincided with both the rise in CRP and the ESR and with refeeding, and its effect size was very small (W = 0.041); no mechanistic interpretation should be built on it. The experimental pro-inflammatory effects of asprosin may operate at the tissue level through paracrine or autocrine mechanisms that are not reflected in circulating concentrations [15,16,18,19]; this explanation is speculative and cannot be tested with our data.

### 4.4. The Erythrocyte Sedimentation Rate

The ESR, a secondary finding, discriminated patients from controls as well as CRP did and peaked later, consistent with the slower kinetics of fibrinogen-driven red-cell aggregation compared with CRP [32]; the only moderate ESR–CRP correlation (ρ = 0.20–0.41) is consistent with evidence that CRP itself does not drive red-cell aggregation, so the two tests carry partly independent information [33]. Unlike in an earlier study using the 1992 Atlanta criteria, in which an ESR above 60 mm/h at 36 h predicted severe AP [34], the ESR was unrelated to severity in our cohort, in which the inflammatory burden was lower (median ESR 24 mm/h in the seven severe cases, compared with a mean of 77 mm/h in that study). ESR values could not be corrected for haematocrit or fibrinogen, which were not measured.

### 4.5. Fasting, Refeeding and Length of Stay

Asprosin is regulated by nutritional state [9,10], and nutritional conditions differed between groups and time points: pre-admission fasting was not recorded, patients were kept nil by mouth for at least 48 h according to the routine practice of our department (longer than the 24–48 h now suggested for mild AP [35]), most had been refed before the 72-h sample, and controls were sampled after an overnight fast. Because inflammation, intravenous fluids, stress and refeeding may affect asprosin in different directions, the net effect of these differences cannot be predicted, and our findings may not apply to centres that practise early feeding.

The nominal inverse associations of asprosin with glucose and with length of stay did not survive correction for multiplicity and were not adjusted for complications, aetiology or intake; they are hypothesis-generating only. One speculative explanation for the association with length of stay is recovery of appetite, given the orexigenic action of asprosin [10].

### 4.6. Clinical Implications

Asprosin AUCs of about 0.57 for moderately severe or severe disease do not support clinical use, although the upper confidence limits (up to 0.71) do not exclude modest discrimination, and with only seven severe cases, the study is uninformative about severe AP. No single marker performed well (the best was age, AUC 0.653). Reporting negative and inconclusive results helps to limit publication bias in the biomarker literature.

### 4.7. Strengths and Limitations

Strengths include prospective serial sampling, severity assessment by the revised Atlanta classification and the modified Marshall score and the separation of pre-specified from post hoc analyses.

The main limitations—confounding by age and body mass index, the exclusion of 55% of screened patients and the lack of in-house assay validation—are discussed in Section 4.1. In addition, the study had 80% power only for a standardised difference of 0.44 (observed 0.24; approximately 271 participants per group would have been needed), and equivalence could not be demonstrated, so a difference of up to medium size is not excluded. Only seven patients had severe AP, and none died, so no conclusion can be drawn for severe disease, and the time of onset of organ failure and complications was not recorded. Time from symptom onset to admission, pre-admission fasting, intake and fluid volumes were not recorded. Metabolic eligibility was defined by laboratory criteria in patients but by recorded diagnoses in controls. Finally, this was a single-centre study in a Turkish population.

## 5. Conclusions

In this exploratory, hypothesis-generating study, serum asprosin was modestly and non-significantly higher in patients with acute pancreatitis than in healthy controls, changed little during the first five days of hospitalisation and showed no statistically significant association with disease severity. Confounding by age and body mass index, the exclusion of patients with diabetes or hypertriglyceridaemia (≥200 mg/dL) and the lack of in-house assay validation preclude a definitive assessment of asprosin as a diagnostic or prognostic marker. The effect estimates reported here can inform the design of larger, age- and body mass index-matched studies with validated assays and recorded nutritional variables.

## Figures and Tables

**Table 1:** Analysis hierarchy and handling of multiplicity.

Multiplicity	Method	Analysis	Level
None; single primary test, α = 0.05	Mann–Whitney U; equivalence by TOST on log-transformed values (margin ±0.5 SD; post hoc)	Admission asprosin, patients (*n* = 80) vs. controls (*n* = 81)	Primary
Bonferroni for pairwise Wilcoxon tests	Friedman test and Kendall W; linear mixed-effects model (random patient intercept)	Change in asprosin over 0, 72 and 120 h (*n* = 80)	Secondary (pre-specified)
Not adjusted; consistency across time points considered	Mann–Whitney U; AUC with bootstrap 95% CI	Asprosin in moderately severe or severe vs. mild AP at each time point; ROC analysis	Secondary (pre-specified)
Not adjusted; supportive only	Rank-based regression; Cohen kappa	Covariate adjustment, log transformation, trimming, age strata; Ranson ≥ 3 as alternative reference standard	Sensitivity (post hoc)
Not adjusted; supportive only	Mann–Whitney U; mixed-model interaction	Exclusion of controls not meeting patient metabolic criteria; time to oral refeeding	Sensitivity (post hoc)
Bonferroni	Kruskal–Wallis, Dunn post hoc	Biliary vs. non-biliary AP vs. controls	Exploratory
Descriptive	Kruskal–Wallis, Jonckheere–Terpstra	Three Atlanta categories, including severe AP (*n* = 7)	Exploratory
Benjamini–Hochberg false discovery rate	Spearman	84 correlations of asprosin with clinical and laboratory variables	Exploratory
None; outside the correlation family	Partial Spearman correlation	Asprosin vs. length of stay adjusted for age, Atlanta category and time to refeeding	Exploratory
None; hypothesis-generating	Matched comparisons; DeLong test	Age/sex-, BMI/sex- and age/BMI/sex-matched subsamples; ESR vs. CRP AUC comparison	Post hoc

AP, acute pancreatitis; AUC, area under the curve; BMI, body mass index; CRP, C-reactive protein; ESR, erythrocyte sedimentation rate; ROC, receiver operating characteristic; SD, standard deviation; TOST, two one-sided tests.

**Table 2:** Demographic, anthropometric and laboratory characteristics of patients with acute pancreatitis and healthy controls at admission.

r (95% CI)	*p*	Controls (*n* = 82)	Patients (*n* = 82)	Variable
0.48 (0.33 to 0.64)	<0.001	42 (32–52)	62 (44–76)	Age, years
—	0.875	35 (42.7)	37 (45.1)	Male sex, *n* (%)
−0.16 (−0.32 to 0.03)	0.085	1.71 (1.68–1.76)	1.69 (1.65–1.75)	Height, m
0.40 (0.23 to 0.55)	<0.001	73 (67–79)	79 (74–83)	Weight, kg
0.61 (0.47 to 0.74)	<0.001	24.67 (22.87–25.90)	27.27 (25.82–28.73)	Body mass index, kg/m^2^
0.35 (0.19 to 0.49)	<0.001	0 (0–6)	8 (0–24)	Smoking, pack-years
0.16 (−0.01 to 0.34)	0.071	3.55 (2.24–4.92)	4.52 (2.94–5.89)	Serum asprosin, ng/mL *
1.00 (1.00 to 1.00)	<0.001	65 (49–79)	1064 (430–2223)	Amylase, U/L
1.00 (1.00 to 1.00)	<0.001	38 (26–52)	1460 (1192–2410)	Lipase, U/L
0.70 (0.57 to 0.81)	<0.001	2.75 (1.42–4.67)	8.75 (5.35–19.22)	C-reactive protein, mg/L
0.62 (0.48 to 0.75)	<0.001	10 (6–12)	19 (11–28)	Erythrocyte sedimentation rate, mm/h
0.74 (0.63 to 0.85)	<0.001	0.62 (0.33–1.04)	2.67 (1.37–5.26)	CRP-to-albumin ratio
−0.77 (−0.87 to −0.66)	<0.001	4.40 (4.12–4.67)	3.68 (3.20–4.00)	Albumin, g/dL
−0.53 (−0.67 to −0.37)	<0.001	7.4 (7.1–7.7)	6.8 (6.3–7.3)	Total protein, g/dL
0.71 (0.58 to 0.83)	<0.001	191 (171–212)	285 (229–408)	Lactate dehydrogenase, U/L
0.64 (0.50 to 0.77)	<0.001	4.40 (3.73–5.42)	7.18 (5.46–10.36)	Neutrophils, ×10^3^/µL
−0.44 (−0.59 to −0.29)	<0.001	2.27 (1.87–2.90)	1.69 (1.12–2.22)	Lymphocytes, ×10^3^/µL
0.69 (0.56 to 0.80)	<0.001	1.85 (1.41–2.43)	4.07 (2.68–8.06)	Neutrophil-to-lymphocyte ratio
0.69 (0.58 to 0.80)	<0.001	23 (18–26)	72 (29–172)	AST, U/L
0.48 (0.32 to 0.63)	<0.001	20 (12–28)	44 (21–176)	ALT, U/L
0.50 (0.34 to 0.65)	<0.001	90 (71–100)	117 (90–162)	ALP, U/L
0.63 (0.50 to 0.76)	<0.001	20 (15–30)	72 (26–238)	GGT, U/L
0.23 (0.05 to 0.40)	0.012	0.78 (0.67–0.89)	0.87 (0.68–1.08)	Creatinine, mg/dL
0.37 (0.21 to 0.53)	<0.001	26.2 (20.1–34.0)	30.4 (26.5–46.4)	Urea, mg/dL
0.09 (−0.09 to 0.27)	0.296	8.99 (8.60–9.30)	9.00 (8.77–9.38)	Albumin-corrected calcium, mg/dL
0.31 (0.14 to 0.47)	<0.001	90 (84–95)	94 (89–101)	Glucose, mg/dL
0.65 (0.51 to 0.78)	<0.001	1.93 (1.69–2.26)	2.74 (2.31–3.42)	HOMA-IR
−0.38 (−0.53 to −0.22)	<0.001	186 (160–214)	159 (135–191)	Total cholesterol, mg/dL
−0.29 (−0.46 to −0.12)	0.001	50.6 (40.4–58.2)	43.0 (34.2–49.0)	HDL cholesterol, mg/dL
−0.18 (−0.35 to −0.00)	0.053	103.8 (89.0–132.3)	93.5 (76.1–121.0)	LDL cholesterol, mg/dL
−0.50 (−0.65 to −0.35)	<0.001	130 (91–183)	89 (61–114)	Triglycerides, mg/dL

Data are the median (interquartile range) unless stated otherwise. *p* values from the Mann–Whitney U test (chi-square test for sex). r, rank-biserial correlation coefficient (positive values indicate higher values in patients) with bootstrap 95% confidence interval (2000 resamples). * Serum asprosin available for 80 patients and 81 controls (insufficient sample volume). Patient values for CRP, the ESR and the CRP-to-albumin ratio refer to the admission (0 h) sample. AST, aspartate aminotransferase; ALT, alanine aminotransferase; ALP, alkaline phosphatase; CRP, C-reactive protein; ESR, erythrocyte sedimentation rate; GGT, gamma-glutamyl transferase; HDL, high-density lipoprotein; HOMA-IR, homeostasis model assessment of insulin resistance; LDL, low-density lipoprotein.

**Table 3:** Serum asprosin and other markers according to disease severity by the revised Atlanta classification. (**A**) Comparison across severity categories. (**B**) Discrimination of moderately severe or severe disease (*n* = 28) from mild disease (*n* = 54).

**(A)**					
***p* (Trend)**	***p* (KW)**	**Severe (*n* = 7)**	**Moderately Severe (*n* = 21)**	**Mild (*n* = 54)**	**Variable**
0.009	0.008	75 (74–86)	69 (50–77)	55 (42–69)	Age, years
—	0.740	4 (57.1)	10 (47.6)	23 (42.6)	Male sex, *n* (%)
—	0.742	4 (57.1)	12 (57.1)	26 (48.1)	Biliary aetiology, *n* (%)
0.726	0.832	27.41 (26.76–29.52)	27.41 (26.42–28.37)	27.18 (25.58–28.96)	Body mass index, kg/m^2^
0.030	0.088	9 (8–10)	8 (7–11)	7 (6–9)	Length of stay, days
0.312	0.494	4.79 (3.31–5.77)	4.70 (3.40–7.01)	3.88 (2.93–5.75)	Asprosin 0 h, ng/mL *
0.272	0.528	4.47 (3.64–6.95)	4.03 (2.85–6.53)	3.48 (2.33–5.77)	Asprosin 72 h, ng/mL *
0.288	0.572	4.83 (3.21–6.88)	4.54 (3.01–6.52)	3.61 (2.39–6.02)	Asprosin 120 h, ng/mL *
0.522	0.813	11.4 (5.9–48.2)	14.0 (5.5–19.8)	8.1 (5.5–18.4)	CRP 0 h, mg/L
0.125	0.282	70.1 (32.0–183.5)	147.2 (21.7–218.0)	54.1 (15.4–114.6)	CRP 72 h, mg/L
0.041	0.107	85.8 (48.1–124.0)	103.0 (10.7–167.0)	29.9 (8.2–77.8)	CRP 120 h, mg/L
0.855	0.703	20 (14–32)	19 (10–22)	20 (11–30)	ESR 0 h, mm/h
0.840	0.960	24 (21–60)	33 (26–42)	30 (21–50)	ESR 72 h, mm/h
0.297	0.587	22 (17–40)	26 (16–37)	22 (15–30)	ESR 120 h, mm/h
0.450	0.506	3.85 (3.07–4.84)	6.14 (2.66–11.36)	3.66 (2.70–6.48)	Neutrophil-to-lymphocyte ratio
0.051	0.104	3.20 (3.00–3.59)	3.40 (3.10–3.90)	3.70 (3.50–4.00)	Albumin, g/dL
0.031	0.073	2 (2–4)	2 (1–3)	2 (0–2)	Ranson score at 48 h
**(B)**					
***p***	**95% CI**	**AUC**		**Marker**	
0.276	0.442–0.707	0.576		Asprosin 0 h **	
0.292	0.439–0.700	0.573		Asprosin 72 h **	
0.299	0.445–0.694	0.572		Asprosin 120 h **	
0.024	0.525–0.771	0.653		Age	
0.528	0.401–0.680	0.543		CRP 0 h	
0.114	0.463–0.744	0.607		CRP 72 h	
0.039	0.511–0.767	0.640		CRP 120 h	
0.736	0.344–0.611	0.477		ESR 0 h	
0.822	0.381–0.651	0.516		ESR 72 h	
0.316	0.428–0.708	0.568		ESR 120 h	
0.038	0.508–0.752	0.638		Ranson score at 48 h	
0.069	0.484–0.761	0.623		Albumin (lower values)	
0.376	0.423–0.690	0.560		Neutrophil-to-lymphocyte ratio	

Data are the median (interquartile range) unless stated otherwise. *p* (KW), Kruskal–Wallis test (chi-square test for categorical variables); *p* (trend), Jonckheere–Terpstra test across ordered categories. Because only seven patients had severe acute pancreatitis, comparisons involving this category are descriptive. * Asprosin available for 80 patients (mild *n* = 54; moderately severe *n* = 19; severe *n* = 7). CRP, C-reactive protein; ESR, erythrocyte sedimentation rate. AUC, area under the receiver operating characteristic curve, with bootstrap 95% confidence interval (2000 resamples); *p* tests the null hypothesis AUC = 0.50 (Mann–Whitney U). For albumin, lower values were treated as indicating higher risk. ** *n* = 80 (26 with moderately severe or severe disease).

## Data Availability

The data supporting the findings of this study are available from the corresponding author upon reasonable request.

## Supplementary Materials

The following supporting information can be downloaded at: https://www.mdpi.com/article/10.3390/diagnostics16213700/s1, Table S1: Sensitivity analyses of the difference in admission serum asprosin between patients and controls; Table S2: Laboratory parameters in biliary acute pancreatitis, non-biliary acute pancreatitis and controls; Table S3: Serum asprosin in non-biliary acute pancreatitis versus controls, unadjusted and adjusted for age and body mass index; Table S4: Comparison of mild with moderately severe or severe acute pancreatitis (revised Atlanta classification); Table S5: Agreement between a Ranson score ≥ 3 at 48 h and the revised Atlanta classification; Table S6: Sensitivity analysis using the Ranson criterion: markers in patients with a Ranson score <3 and ≥3 at 48 h; Table S7: Spearman correlations between serum asprosin and clinical and laboratory variables in patients (84 correlations); Table S8: Serum asprosin and indicators of disease severity (Spearman correlation); Table S9: Spearman correlations between serum asprosin and clinical and laboratory variables in healthy controls (*n* = 81); Table S10: Linear mixed-effects models of serial serum asprosin and the effect of time to oral refeeding (*n* = 80 patients, 240 observations); Table S11: Unadjusted and adjusted effect estimates for the difference in admission serum asprosin between patients and controls, including matched analyses; Figure S1: Receiver operating characteristic curves for moderately severe or severe acute pancreatitis (*n* = 28) versus mild disease (*n* = 54).

## Abbreviations

AP	Acute pancreatitis
AUC	Area under the curve
CI	Confidence interval
CRP	C-reactive protein
ELISA	Enzyme-linked immunosorbent assay
ERCP	Endoscopic retrograde cholangiopancreatography
ESR	Erythrocyte sedimentation rate
HbA1c	Glycated haemoglobin
HOMA-IR	Homeostasis model assessment of insulin resistance
IKKβ	IκB kinase beta
LDL	Low-density lipoprotein
NF-κB	Nuclear factor kappa B
NLR	Neutrophil-to-lymphocyte ratio
NLRP3	NLR family pyrin domain containing 3
ROC	Receiver operating characteristic
STARD	Standards for Reporting Diagnostic Accuracy Studies
STROBE	Strengthening the Reporting of Observational Studies in Epidemiology
TOST	Two one-sided tests
