# Supplementary material for: Serum Asprosin Concentrations and Severity in Acute Pancreatitis: A Prospective Case–Control Study with Serial Measurements
Source: Diagnostics (Basel). 2026 Nov 9;16(21):3700. doi: 10.3390/diagnostics16213700 (PMC13763098; doi:10.3390/diagnostics16213700)
Supplement: Supplementary file 1 [file diagnostics-16-03700-s001.zip › diagnostics-4620894-supplementary.pdf]

Supplementary Material

Serum asprosin concentrations and severity in acute pancreatitis: a prospective case–control study with serial measurements

Table S1. Sensitivity analyses of the difference in admission serum asprosin between patients and controls.

Analysis	n (patients / controls)	Effect estimate	P
Unadjusted (Mann–Whitney U)	80 / 81	r = 0.16	0.071
Adjusted for age (rank-based regression)	80 / 81	β = 13.3 ranks	0.099
Adjusted for body mass index (rank-based regression)	80 / 81	β = 16.1 ranks	0.062
Adjusted for age and body mass index (rank-based regression)	80 / 81	β = 15.8 ranks	0.078
Adjusted for age, body mass index and sex (rank-based regression)	80 / 81	β = 14.0 ranks	0.113
Group × age interaction (rank-based regression)	80 / 81	—	0.162
Log-transformed values (t test)	80 / 81	Cohen d = 0.24	0.128
Extreme values (> 3 × IQR) removed	74 / 77	r = 0.16	0.091
Controls not meeting patient metabolic criteria excluded*	80 / 57	r = 0.18	0.076
Equivalence, TOST on log scale (margin ±0.5 SD)	80 / 81	d = 0.24 (90% CI −0.02 to 0.50)	0.051
1:1 age- and sex-matched (age ± 3 years)†	46 pairs	Median paired difference 1.91 ng/mL (95% CI 0.24 to 3.78)	0.022
1:1 BMI- and sex-matched (BMI ± 1 kg/m²)†	45 pairs	Median paired difference 0.96 ng/mL (95% CI −0.34 to 2.72)	0.150

*Controls with fasting glucose ≥ 100 mg/dL or triglycerides ≥ 200 mg/dL (n = 25) excluded. †Optimal 1:1 matching without replacement with exact sex matching; Hodges–Lehmann median paired difference and paired Wilcoxon signed-rank test; the age-, BMI- and sex-matched analysis is given in Table S11. Rank-based regression: ordinary least squares on ranked asprosin with ranked continuous covariates; β, adjusted difference in mean rank (patients − controls). TOST, two one-sided tests; IQR, interquartile range; BMI, body mass index; SD, standard deviation.

Table S2. Laboratory parameters in biliary acute pancreatitis, non-biliary acute pancreatitis and controls.

Variable	Biliary (n = 42)	Non-biliary (n = 40)	Controls (n = 82)	P (KW)	B vs NB	B vs C	NB vs C
Age, years	64 (52–77)	58 (35–74)	42 (32–52)	<0.001	0.161	<0.001	0.004
Body mass index, kg/m²	28.22 (26.88–29.38)	26.92 (25.20–27.96)	24.67 (22.87–25.90)	<0.001	0.047	<0.001	<0.001
Asprosin 0 h, ng/mL	4.29 (2.49–5.49)	4.62 (3.11–6.59)	3.55 (2.24–4.92)	0.128	1.000	1.000	0.135
Asprosin 72 h, ng/mL	3.01 (2.33–4.73)	4.67 (3.08–6.55)	3.55 (2.24–4.92)	0.078	0.129	1.000	0.136
Asprosin 120 h, ng/mL	3.30 (2.46–5.41)	4.78 (3.10–7.95)	3.55 (2.24–4.92)	0.080	0.192	1.000	0.102
CRP 0 h, mg/L	7.35 (4.35–17.40)	10.85 (6.70–31.83)	2.75 (1.42–4.67)	<0.001	0.863	<0.001	<0.001
ESR 0 h, mm/h	17 (10–28)	21 (14–30)	10 (6–12)	<0.001	0.477	<0.001	<0.001
AST, U/L	115 (70–222)	34 (23–87)	23 (18–26)	<0.001	0.012	<0.001	<0.001
ALT, U/L	81 (40–188)	24 (14–78)	20 (12–28)	<0.001	<0.001	<0.001	0.106
ALP, U/L	127 (92–194)	113 (89–149)	90 (71–100)	<0.001	1.000	<0.001	<0.001
GGT, U/L	120 (64–290)	38 (19–79)	20 (15–30)	<0.001	0.002	<0.001	<0.001
Amylase, U/L	1268 (549–2792)	932 (350–1920)	65 (49–79)	<0.001	1.000	<0.001	<0.001
Lipase, U/L	1455 (1242–2410)	1465 (722–2295)	38 (26–52)	<0.001	1.000	<0.001	<0.001
Albumin-corrected calcium, mg/dL	9.00 (8.80–9.50)	9.02 (8.71–9.20)	8.99 (8.60–9.30)	0.412	1.000	0.551	1.000

Data are median (interquartile range). P (KW), Kruskal–Wallis test; pairwise columns give Bonferroni-corrected Dunn test P values. Patient asprosin at 72 h and 120 h is compared with the single control value; these comparisons are descriptive. Asprosin available for 41 biliary and 39 non-biliary patients and 81 controls. B, biliary; NB, non-biliary; C, controls; CRP, C-reactive protein; ESR, erythrocyte sedimentation rate; AST, aspartate aminotransferase; ALT, alanine aminotransferase; ALP, alkaline phosphatase; GGT, gamma-glutamyl transferase.

Table S3. Serum asprosin in non-biliary acute pancreatitis versus controls, unadjusted and adjusted for age and body mass index.

Time point	Unadjusted P	Adjusted for age	Adjusted for BMI	Adjusted for age and BMI
Asprosin 0 h	0.044	0.096	0.099	0.139
Asprosin 72 h	0.044	0.115	0.140	0.202
Asprosin 120 h	0.031	0.101	0.079	0.135

Unadjusted P from the Mann–Whitney U test; adjusted P from rank-based regression (ranked asprosin, ranked covariates). Non-biliary patients were older (mean 54.6 versus 42.3 years) and had a higher body mass index than controls. BMI, body mass index.

Table S4. Comparison of mild with moderately severe or severe acute pancreatitis (revised Atlanta classification).

**Variable**	**Mild (n = 54)**	**Moderately severe or severe (n = 28)**	**r**	**P**
Age, years	55 (42–69)	73 (57–77)	0.31	0.024
Body mass index, kg/m²	27.18 (25.58–28.96)	27.41 (26.53–28.67)	0.04	0.799
Length of stay, days	7 (6–9)	8 (7–11)	0.29	0.028
Ranson score at 48 h	2 (0–2)	2 (1–3)	0.28	0.038
Asprosin 0 h, ng/mL	3.88 (2.93–5.75)	4.74 (3.32–6.32)	0.15	0.276
Asprosin 72 h, ng/mL	3.48 (2.33–5.77)	4.42 (2.93–6.72)	0.15	0.292
Asprosin 120 h, ng/mL	3.61 (2.39–6.02)	4.55 (3.00–7.22)	0.14	0.299
CRP 0 h, mg/L	8.1 (5.5–18.4)	12.7 (5.3–19.9)	0.09	0.528
CRP 72 h, mg/L	54.1 (15.4–114.6)	96.0 (21.8–224.8)	0.21	0.114
CRP 120 h, mg/L	29.9 (8.2–77.8)	94.4 (11.5–152.8)	0.28	0.039
ESR 0 h, mm/h	20 (11–30)	19 (11–26)	−0.05	0.736
ESR 72 h, mm/h	30 (21–50)	32 (24–46)	0.03	0.822
ESR 120 h, mm/h	22 (15–30)	25 (16–38)	0.14	0.316
NLR	3.66 (2.70–6.48)	4.65 (2.70–10.65)	0.12	0.376
Albumin, g/dL	3.70 (3.50–4.00)	3.40 (3.00–3.90)	−0.25	0.069
LDH, U/L	298 (230–431)	282 (228–360)	−0.10	0.481
Glucose, mg/dL	94 (89–99)	96 (89–119)	0.19	0.154
Urea, mg/dL	31.7 (27.0–44.4)	30.1 (25.8–52.6)	0.02	0.880

Data are median (interquartile range); Mann–Whitney U test; r, rank-biserial correlation (positive values indicate higher values in moderately severe or severe disease). Asprosin available in 54 mild and 26 moderately severe or severe cases. CRP, C-reactive protein; ESR, erythrocyte sedimentation rate; LDH, lactate dehydrogenase; NLR, neutrophil-to-lymphocyte ratio.

Table S5. Agreement between a Ranson score ≥ 3 at 48 h and the revised Atlanta classification (n = 82).

Atlanta outcome	TP / FP / FN / TN	Agreement, %	Kappa (95% CI)	P	Sens., %	Spec., %	PPV, %	NPV, %
Moderately severe or severe (n = 28)	12 / 11 / 16 / 43	67.1	0.235 (−0.001 to 0.471)	0.032	42.9	79.6	52.2	72.9
Severe only (n = 7)	3 / 20 / 4 / 55	70.7	0.080 (−0.230 to 0.389)	0.362	42.9	73.3	13.0	93.2

A Ranson score ≥ 3 was treated as the test and the revised Atlanta category as the reference. Ranson scores were available for all 82 patients (23 with a score ≥ 3). TP, true positive; FP, false positive; FN, false negative; TN, true negative; Sens., sensitivity; Spec., specificity; PPV and NPV, positive and negative predictive values.

Table S6. Sensitivity analysis using the Ranson criterion: markers in patients with a Ranson score < 3 and ≥ 3 at 48 h.

**Variable**	**Ranson < 3 (n = 59)**	**Ranson ≥ 3 (n = 23)**	**r**	**P**
Asprosin 0 h, ng/mL	4.52 (2.94–5.76)	4.58 (2.98–6.15)	0.05	0.738
Asprosin 72 h, ng/mL	3.70 (2.36–5.81)	4.64 (2.70–7.59)	0.15	0.316
Asprosin 120 h, ng/mL	3.75 (2.46–5.54)	4.67 (3.00–8.25)	0.16	0.284
NLR	3.42 (2.36–5.50)	6.66 (5.11–13.46)	0.58	< 0.001
CRP 0 h, mg/L	8.2 (4.8–16.3)	14.0 (6.2–50.0)	0.23	0.102
CRP 72 h, mg/L	36.9 (12.3–83.6)	164.1 (73.3–249.2)	0.49	< 0.001
CRP 120 h, mg/L	24.6 (7.6–71.0)	121.2 (59.1–146.9)	0.50	< 0.001
ESR 72 h, mm/h	30 (20–47)	34 (28–55)	0.20	0.165
Age, years	55 (36–71)	74 (64–80)	0.48	< 0.001

Data are median (interquartile range); Mann–Whitney U test; r, rank-biserial correlation. CRP, C-reactive protein; ESR, erythrocyte sedimentation rate; NLR, neutrophil-to-lymphocyte ratio.

Table S7. Spearman correlations between serum asprosin and clinical and laboratory variables in patients (84 correlations).

Variable	ρ 0 h	P	q	ρ 72 h	P	q	ρ 120 h	P	q
Age	−0.104	0.356	0.732	−0.114	0.314	0.732	0.018	0.877	0.972
Body mass index	−0.218	0.052	0.400	−0.205	0.068	0.422	−0.254	0.023	0.319
Length of stay	−0.273	0.014	0.240	−0.238	0.033	0.365	−0.273	0.014	0.240
Ranson score 48 h	−0.015	0.894	0.972	0.019	0.864	0.972	0.029	0.800	0.972
CRP (same time point)	0.080	0.483	0.802	0.112	0.323	0.732	0.054	0.637	0.907
ESR (same time point)	−0.109	0.334	0.732	−0.146	0.197	0.727	−0.117	0.303	0.732
CRP-to-albumin ratio (same time point)	0.097	0.393	0.732	0.135	0.232	0.732	0.075	0.507	0.804
Albumin	−0.113	0.318	0.732	−0.099	0.383	0.732	−0.220	0.050	0.400
Total protein	−0.203	0.070	0.422	−0.177	0.115	0.570	−0.199	0.077	0.431
LDH	−0.026	0.821	0.972	−0.130	0.250	0.732	−0.129	0.255	0.732
Lymphocytes	0.075	0.508	0.804	0.065	0.568	0.838	0.073	0.520	0.809
Neutrophils	0.027	0.809	0.972	0.047	0.678	0.934	0.070	0.539	0.823
NLR	−0.014	0.903	0.972	−0.004	0.971	0.982	0.020	0.863	0.972
AST	0.180	0.111	0.570	0.096	0.399	0.732	0.052	0.650	0.909
ALT	0.120	0.288	0.732	0.016	0.889	0.972	0.010	0.933	0.982
ALP	0.068	0.549	0.823	0.006	0.956	0.982	0.024	0.832	0.972
GGT	0.099	0.384	0.732	−0.030	0.792	0.972	0.003	0.982	0.982
Amylase	0.032	0.781	0.972	0.007	0.951	0.982	0.033	0.774	0.972
Lipase	0.156	0.166	0.717	0.166	0.142	0.662	0.154	0.174	0.717
Creatinine	0.105	0.353	0.732	0.136	0.227	0.732	0.079	0.487	0.802
Glucose	−0.231	0.039	0.365	−0.235	0.036	0.365	−0.295	0.008	0.222
Urea	0.118	0.295	0.732	0.152	0.179	0.717	0.208	0.064	0.422
Corrected calcium	−0.113	0.317	0.732	−0.145	0.199	0.727	−0.091	0.422	0.738
HDL cholesterol	−0.030	0.788	0.972	−0.023	0.838	0.972	−0.056	0.622	0.900
LDL cholesterol	−0.098	0.387	0.732	−0.117	0.299	0.732	−0.034	0.764	0.972
Triglycerides	0.095	0.401	0.732	0.005	0.962	0.982	0.128	0.258	0.732
Total cholesterol	−0.087	0.443	0.759	−0.097	0.391	0.732	−0.034	0.768	0.972
HOMA-IR	0.329	0.003	0.162	0.092	0.420	0.738	0.320	0.004	0.162

Asprosin at each time point was correlated with each variable (n = 80); CRP, the ESR and the CRP-to-albumin ratio refer to the same time point, all other variables to admission. q, Benjamini–Hochberg false discovery rate-adjusted P across all 84 correlations. No correlation remained significant after adjustment (all q ≥ 0.16).

Table S8. Serum asprosin and indicators of disease severity (Spearman correlation).

Indicator	ρ 0 h	P	ρ 72 h	P	ρ 120 h	P
Revised Atlanta category (1–3)	0.112	0.321	0.125	0.270	0.119	0.293
Persistent organ failure > 48 h	0.009	0.940	0.093	0.412	0.070	0.538
Local complication	0.060	0.598	0.046	0.683	0.045	0.695
Systemic complication	−0.064	0.574	−0.015	0.898	0.010	0.929
Worst PaO_2/FiO_2 ratio, first 48 h	−0.066	0.563	0.008	0.944	−0.107	0.346
Maximum creatinine, first 48 h	0.056	0.622	0.107	0.347	0.072	0.529
Ranson score at 48 h	−0.015	0.894	0.019	0.864	0.029	0.800

n = 80. Binary indicators coded 0/1. PaO_2/FiO_2, ratio of arterial oxygen tension to inspired oxygen fraction.

Table S9. Spearman correlations between serum asprosin and clinical and laboratory variables in healthy controls (n = 81).

Variable	ρ	P	q
Age	0.166	0.139	0.486
Body mass index	0.116	0.303	0.606
CRP	−0.103	0.361	0.670
ESR	−0.083	0.462	0.801
CRP-to-albumin ratio	−0.138	0.219	0.549
Albumin	0.290	0.009	0.226
Total protein	0.042	0.710	0.886
LDH	0.219	0.050	0.433
Lymphocytes	−0.016	0.886	0.886
Neutrophils	0.030	0.787	0.886
NLR	−0.039	0.733	0.886
AST	0.165	0.140	0.486
ALT	0.134	0.232	0.549
ALP	0.157	0.162	0.486
GGT	0.155	0.168	0.486
Amylase	−0.025	0.826	0.886
Lipase	0.023	0.839	0.886
Creatinine	0.235	0.034	0.433
Glucose	0.067	0.552	0.845
Urea	0.187	0.095	0.486
Corrected calcium	0.158	0.160	0.486
HDL cholesterol	−0.068	0.545	0.845
LDL cholesterol	−0.040	0.725	0.886
Triglycerides	0.128	0.255	0.553
Total cholesterol	−0.017	0.881	0.886
HOMA-IR	0.039	0.733	0.886

q, Benjamini–Hochberg false discovery rate-adjusted P across the 26 correlations. No correlation remained significant after adjustment.

Table S10. Linear mixed-effects models of serial serum asprosin and the effect of time to oral refeeding (n = 80 patients, 240 observations).

Model	Term	Estimate (95% CI)	P
Model 1: time	72 h vs 0 h	-9.8 (-18.7 to −0.9)	0.032
Model 1: time	120 h vs 0 h	-6.0 (-15.0 to 2.9)	0.187
Model 1: time	Overall effect of time (likelihood ratio, 2 df)	χ^2 = 4.64	0.098
Model 2: time × refeeding	72 h × refeeding at 72 h	-6.6 (-26.0 to 12.9)	0.507
Model 2: time × refeeding	120 h × refeeding at 72 h	2.9 (-16.5 to 22.4)	0.767
Model 2: time × refeeding	Interaction (likelihood ratio, 2 df)	χ^2 = 0.96	0.617
Change 0–72 h by refeeding time	Refed at 48 h (n = 56) vs 72 h (n = 24)	−0.34 vs −0.43 ng/mL	0.883

Outcome: rank-transformed asprosin concentration; random intercept for each patient; maximum likelihood estimation. Estimates are differences in mean rank. Refeeding at 72 h versus 48 h. Change 0–72 h compared with the Mann–Whitney U test.

Table S11. Unadjusted and adjusted effect estimates for the difference in admission serum asprosin between patients and controls, including matched analyses.

Analysis	n (patients / controls)	Estimate (95% CI)	P	Scale
**A. Full sample**				
Hodges–Lehmann median difference (Mann–Whitney U)	80 / 81	0.70 (−0.06 to 1.42)	0.071	ng/mL
Geometric mean ratio, unadjusted	80 / 81	1.21 (0.95 to 1.56)	0.128	ratio
Geometric mean ratio, adjusted for age and sex	80 / 81	1.23 (0.93 to 1.63)	0.154	ratio
Geometric mean ratio, adjusted for BMI and sex	80 / 81	1.15 (0.85 to 1.56)	0.364	ratio
Geometric mean ratio, adjusted for age, BMI and sex	80 / 81	1.17 (0.85 to 1.61)	0.324	ratio
**B. Excluding metabolically ineligible controls (fasting glucose ≥ 100 mg/dL or triglycerides ≥ 200 mg/dL)**				
Hodges–Lehmann median difference (Mann–Whitney U)	80 / 57	0.79 (−0.10 to 1.61)	0.076	ng/mL
Geometric mean ratio, unadjusted	80 / 57	1.26 (0.96 to 1.67)	0.099	ratio
Geometric mean ratio, adjusted for age, BMI and sex	80 / 57	1.23 (0.85 to 1.77)	0.274	ratio
**C. Post hoc 1:1 optimal matching (median paired difference, Wilcoxon signed-rank test)**				
Age (± 3 years) and sex. Median age 46 vs 46 years; median BMI 27.2 vs 24.5 kg/m²	46 pairs	1.91 (0.24 to 3.78)	0.022	ng/mL
BMI (± 1 kg/m²) and sex. Median age 63 vs 44 years; median BMI 26.4 vs 25.9 kg/m²	45 pairs	0.96 (−0.34 to 2.72)	0.150	ng/mL
Age, BMI and sex. Median age 44 vs 43 years; median BMI 25.6 vs 25.9 kg/m²	23 pairs	2.27 (0.05 to 5.21)	0.042	ng/mL

Geometric mean ratios (patients to controls) are from linear regression of log-transformed asprosin with heteroscedasticity-robust (HC3) standard errors. Differences are patients minus controls. Matched pairs were formed by optimal 1:1 matching without replacement within sex. BMI, body mass index; CI, confidence interval.

Figure S1. Receiver operating characteristic curves for moderately severe or severe acute pancreatitis (n = 28) versus mild disease (n = 54).

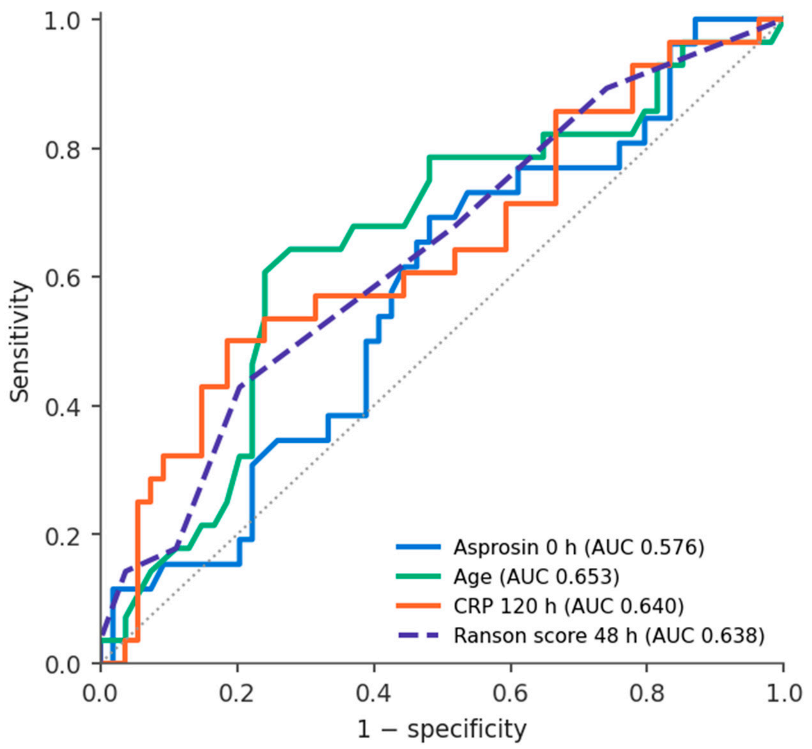

Asprosin at admission (n = 80), age, C-reactive protein (CRP) at 120 h and the Ranson score at 48 h. AUC, area under the curve.
